# PLOS Currents: Outbreaks --- For findings that the world just can't wait to see

**DOI:** 10.1371/currents.outbreaks.8ed218c079fbded60c505f025ed45f67

**Published:** 2013-05-15

**Authors:** Ben Cowling, Jean-Claude Desenclos, Steven Riley, Lone Simonsen, Cecile Viboud

**Affiliations:** University of Hong Kong; Directeur scientifique at Institut de Veille Sanitaire (InVS), Saint Maurice, France; School of Public Health, Imperial College London, London, UK; Research Professor at Department of Global Health, George Washington University, School of Public Health and Health Services, DC, Bethesda, Maryland; Research scientist, US National Institutes of Health at Fogarty International Center, National Institutes of Health, Bethesda, MD, USA

## Editorial

There are times in research when speed really is of the essence in getting results published. For those working in the infectious disease field, there is a particularly pressing need when an outbreak occurs and it becomes paramount to get data communicated both efficiently and promptly.

The 2009 influenza pandemic highlighted the limited options available for scholarly publications to rapidly communicate research findings in a time of crisis. In response to the needs of the community, PLOS launched PLOS Currents: Influenza as a publication channel that would meet the need for swift publication of research during that influenza pandemic. PLOS Currents: Influenza allowed the process of peer review and publication to be reduced from months to a matter of days.

PLOS Currents: Influenza showed that agile and reactive publication avenues that facilitated the rapid exchange of scientific results and ideas during an emergency period were possible (since then we have launched another section, PLOS Currents: Disasters, where timely publication is also of the essence). We are now using the experience from PLOS Currents: Influenza to benefit other research areas that are also subject to outbreaks and pandemics, and with this in mind, we are re-launching PLOS Currents: Influenza as PLOS Currents: Outbreaks, an expanded section with a broader scope that will consider research in all aspects of infectious disease outbreaks.

PLOS Currents: Outbreaks aims to provide a focused venue for the publication of breaking research in the time of a major outbreak or a pandemic. PLOS Currents: Outbreaks will offer a channel to communicate work in any area of research and public health relevant to infectious diseases outbreaks with impact on human health and provide a service to the community in times of crisis. PLOS Currents: Outbreaks will have a broad scope covering field investigations, serological and epidemiological studies, mathematical models intended to support pandemic decision making, research into vaccine development and vaccination programs, cell biology and genomics studies into infectious agents, and pieces covering policy and social science matters with relevance to infectious disease outbreaks.

Work published by PLOS Current: Outbreaks will undergo formal rapid peer-review, either by members of the Reviewer Board or by external reviewers. We hope that the scope and clarity of submissions to PLOS Currents: Outbreaks will greatly facilitate a swift review process. We are looking for manuscripts that address a single clearly stated objective and that present evidence that can be robustly reviewed in a short time. Although we anticipate that many submissions will be based on publicly available data, analyzed with standard techniques, the need for rapid review won’t necessarily preclude the use of more sophisticated methods. However, the trail of evidence supporting the use of techniques such as mechanistic models and novel inference methods will need to be clear, perhaps drawing on closely related prior publications or freely available, and easily executable source code. It seems unlikely that articles relying on extensive supporting information will meet the objectives of the journal.

PLOS Currents: Outbreaks therefore encourages submissions that report ongoing and first-cut analysis of emerging outbreaks; it allows the timely publication of work that would otherwise go uncredited or lose its cutting-edge relevance – for example the sequence of a new pathogen where the set of analyses that would allow the preparation of a traditional research paper has not been completed.

Articles published at PLOS Currents: Outbreaks will immediately become part of the global scientific record. They will be registered with PubMed and count as a peer reviewed publication. PLOS Currents: Outbreaks will thus allow a venue to publish ongoing research rapidly, while leaving the door open for authors to submit a full paper elsewhere once a longer, more comprehensive manuscript has been completed (in full compliance with all applicable publication terms and conditions, including a transparent acknowledgment of work already reported in a preliminary form).

An example of how we envisage the process of publication of relevant evidence for infectious disease outbreaks is reflected by this article in PLOS Currents: Influenza, that was followed by the publication of a more in-depth analysis in a traditional journal.

The scope and format of the submission will allow a swift peer review which aims for publication of sound research within two to three weeks from submission. The peer review process will be focused solely on an assessment of the soundness of the work and on establishing whether or not the piece is suitable for publication; therefore, request for revisions to the submitted piece will be kept to a minimum.

As for all PLOS publications, articles published in all PLOS Currents sections are open access under a CC-BY license. However, unlike many other open access publications, PLOS Currents: Outbreaks has no publication charge.

We are certain that PLOS Currents: Outbreaks will address a need in scholarly publishing and provide a channel for the publication of breaking research when it is most needed. We look forward to working with researchers in these rapidly moving fields.

If you are interested in submitting work to PLOS Currents: Outbreaks, in joining our Board of Reviewers or in supporting this initiative please contact us on currents@plos.org.

